# Long‐Term Evolution Under Heatwave Conditions in the Seed Beetle, *Callosobruchus maculatus*

**DOI:** 10.1002/ece3.73562

**Published:** 2026-04-23

**Authors:** Edward R. Ivimey‐Cook, Sarah Glavan, Sophie Bricout, Claudio Piani, Elena C. Berg

**Affiliations:** ^1^ School of Biological Sciences University of East Anglia Norwich UK; ^2^ The American University of Paris Paris France

**Keywords:** *Callosobruchus maculatus*, climate change, fitness, fluctuating temperatures, heatwaves, thermal adaptation

## Abstract

Heatwaves, temporary periods of elevated temperatures, are increasing in both magnitude and frequency and have devastating effects on many taxa. However, to date, most studies investigating the impacts of heatwaves have focused on populations that have evolved under constant conditions prior to assaying or have only investigated the short‐term outcomes. Here, using the seed beetle, *Callosobruchus maculatus* , we investigated the long‐term effects of evolution after 43 generations of fluctuating temperature with added heatwave exposure (a +2°C increase peaking at 42°C across 7 days, once every generation) on two important life history traits, development time and lifetime reproductive success (LRS). We find that populations that evolved under heatwave conditions eclosed at similar times but had lower LRS than those that evolved and were assayed under fluctuating conditions. When assayed at a novel constant and benign temperature of 29°C, beetles from both thermal regimes developed slower but had similar LRS. Together, this suggests that long‐term heatwave exposure may incur only a very small cost to fitness, which disappears when individuals from those populations are exposed to benign control conditions. This study emphasizes the potency of long‐term multigenerational exposure to heatwaves in order to understand how populations respond to climate change.

## Graphical abstract

Here, using the seed beetle, *Callosobruchus maculatus*, we investigated the long‐term effects of evolution after 43 generations of fluctuating temperature with added heatwave exposure on two important life history traits, development time and lifetime reproductive success (LRS). We find that long‐term heatwave exposure resulted in little cost to the life history of the beetle, whereas exposure to benign conditions resulted in maximal performance.

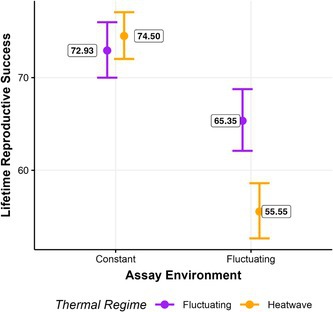

## 1 Introduction

Natural populations are responding to global temperature increases by shifting ranges, declining in abundance, or ultimately, going extinct (Parmesan and Yohe 2003; Thomas et al. 2004; Chen et al. 2011). Not only are average annual temperatures rising, but extreme climatic events, such as heatwaves, are becoming far more frequent (Wang et al. 2024). Heatwaves, defined as three or more consecutive days of temperature above the 90th percentile for each calendar day (Perkins and Alexander 2013), have been shown to negatively affect the persistence and adaptation of natural populations (Stillman 2019; Ma et al. 2021; Murali et al. 2023), including plant and animal life (Smith 2011; Smale and Wernberg 2013), with broad‐scale simultaneous effects on marine and terrestrial ecosystems (Ruthrof et al. 2018). These effects are particularly alarming given that heatwaves are predicted to continue to increase across all scales and impact all inhabited regions of the globe (Perkins‐Kirkpatrick and Lewis 2020; Intergovernmental Panel on Climate Change (IPCC) 2023).

The majority of animal or evolutionary research investigating the effects of heatwaves has been conducted under controlled laboratory conditions. Such studies have been particularly helpful in highlighting the devastating impact that exposure to extreme elevated temperatures can have on organismal function. For instance, exposure to periods of persistent elevated temperature has been shown to reduce growth and biomass in plants and reduce reproductive success and increase mortality in a wide variety of animals. In the two tree species *Pinus taeda* and *Quercus rubra* , monthly heatwaves of +12°C significantly reduced total growth as well as leaf, stem and root biomass (Bauweraerts et al. 2012). In male stickleback fish (*Gasterosteus aculeatus*), short‐term heatwaves at 23°C for 5 days were found to suppress parental care, delay hatching, reduce hatching success, and negatively impact offspring body condition and swimming performance (Barrett and Stein 2024). In the zebra finch (*Taeniopygia guttata*), exposure of males to 30°C or 40°C temperatures daily for 14 consecutive days led to an increase in cloacal temperature and a reduction in the proportion of sperm with normal morphology (Hurley et al. 2018).

In insects, extreme high temperatures (EHTs) are known to alter fitness‐related life history traits such as survival, development, and reproduction (Ma et al. 2021). Among these traits, reproductive potential is predicted to be the most susceptible to impacts from EHTs (Zhang et al. 2015; Walsh et al. 2019). The fruit fly, *Drosophila melanogaster* , is known to display reproductive sensitivity to temperature, with over half of males (above 50% median) becoming sterile when temperatures exceed 30°C (Rohmer et al. 2004; David et al. 2005). In the flour beetle, *Tribolium castaneum* , experimental heatwave conditions reduce male fertility and sperm competitiveness, resulting in reduced reproduction and a decrease in the lifespan of offspring (Sales et al. 2018).

Most laboratory studies investigating the impact of heatwaves have used populations that have evolved under constant temperature conditions rather than exposing them to realistic daily temperature fluctuations (Hurley et al. 2018; Sales et al. 2018, 2024; Breedveld et al. 2023; Barrett and Stein 2024; although see Weaving et al. 2024). Fortunately, the need to consider the effects of thermal fluctuation is now well documented (Vasseur et al. 2014; Colinet et al. 2015; Sinclair et al. 2016; Bagni et al. 2024), and a number of studies have incorporated realistic thermal regimes into experimental designs, including changes to both the mean and variability of temperatures (Hokanson et al. 1977; Niehaus et al. 2012; Paaijmans et al. 2013; Vasseur et al. 2014; Bozinovic et al. 2016; Matsubara 2018; Schaum et al. 2018, 2022; Buckley and Kingsolver 2021; but see Bagni et al. 2024). For instance, a study by Weaving et al. (2024) investigated the effects of heatwaves on male and female tsetse flies (*Glossina pallidipes*) by exposing the flies to fluctuating temperatures peaking at 36°C, 38°C, or 40°C for 2 h. At all heatwave temperatures, males and females experienced equivalent fertility loss. At 38°C in particular, the combination of declining mortality and fertility resulted in a 10.8% population decline compared to the control treatment. In contrast, 40°C resulted in 100% mortality of individuals. Despite this increasing emphasis on realistic environmental conditions, there is still a significant lack of data on the long‐term evolutionary impacts of exposure to heatwaves (Gutschick and BassiriRad 2003; van de Pol et al. 2017), with most studies exposing individuals to short blasts of elevated temperature. This underscores the need to study the long‐term evolution of populations exposed to heatwaves in combination with realistic diel fluctuations, particularly as recent evidence suggests that populations evolving under realistic fluctuating conditions may end up better able to cope with both short‐ and long‐term changes in environmental conditions (Ivimey‐Cook et al. 2024).

Here, we investigated the effect of long‐term exposure to heatwaves on the lifetime reproductive success (hereafter, LRS) and development time (from egg to adult eclosion) of the seed beetle, *Callosobruchus maculatus* , after multiple generations of evolving under realistic fluctuating conditions in a laboratory setting. In a previous experiment, we examined the impacts of short‐ and long‐term exposure to fluctuating temperatures (with no exposure to heatwaves) on *C. maculatus* life history (Ivimey‐Cook et al. 2024). Briefly, we found that evolving within a fluctuating environment leads to increased reproductive performance upon exposure to constant conditions. In contrast, we found no difference in LRS between those that evolved and were assayed under fluctuating or constant conditions (i.e., experienced no change in environment). In this follow‐up study, we exposed the same population of beetles to long‐term heatwave exposure. Although evolving under realistic diel fluctuations can promote a broadening of thermal niche (Ivimey‐Cook et al. 2024), we might predict that exposure to short‐term extreme heat will ultimately contribute to a reduction in reproductive fitness and impaired development (Sales et al. 2018, 2021; Weaving et al. 2024). Alternatively, beetle populations exposed to long‐term repeated heatwaves might exhibit improved tolerance and resistance to elevated temperatures, resulting in reproductive fitness and rates of development that are similar to populations evolving under purely fluctuating temperatures (French et al. 2019; Ahrens et al. 2021; Xu et al. 2021). Furthermore, as shown in Ivimey‐Cook et al. (2024) and Ketola et al. (2013), evolving under fluctuating conditions may have beneficial effects on reproductive performance when individuals are exposed to a constant environment. We may therefore expect a similar increase in fitness upon exposure to a constant environment for those that evolved under fluctuating conditions with repeated heatwaves.

## 2 Methods

### 2.1 Study System

*Callosobruchus maculatus* is a globally widespread agricultural pest native to Africa and Asia (Beck and Blumer 2014). Females of this species deposit their eggs on the surface of dried legumes, such as mung beans ( *Vigna radiata* ) or black‐eyed beans ( *Vigna unguiculata* ). Upon hatching, the larvae burrow into the bean, eclosing as adults 21–27 days later. Adult seed beetles are facultatively aphagous, obtaining all necessary resources during their larval stage within the bean. Adults without food or water can live up to 2 weeks, while adults with access to nutrients can live 3 weeks or more (Fox 1993; Ursprung et al. 2009). As soon as they emerge from their beans, adult females of this species can start mating and laying eggs immediately (Beck and Blumer 2014).

In this experiment, we used a strain of *C. maculatus* originating from South India (SI). The original SI beetles were collected in Tirunelveli, India, in 1979 (Mitchell 1991) and were subsequently raised at the University of Kentucky USA (hereafter referred to as SI USA). We obtained this strain from Uppsala University in Sweden in 2015 and have since maintained it at the American University of Paris. Stock populations have been cultured exclusively on mung beans and kept in climate chambers at a constant 29°C, 50% relative humidity, and a 12:12 h light: dark cycle. The stock populations have been kept at these standard laboratory conditions for decades (Berger et al. 2014).

Prior to the experiment, beetles were kept in one liter jars containing 250 g of beans, and approximately 250–350 newly hatched beetles were moved to fresh jars with beans every 24 days. Adequate beans were provided to allow each female to lay just one egg per bean, thus preventing competition among multiple larvae within a single bean (Berg and Maklakov 2012; Berg et al. 2019). This is important as larval competition has been found to significantly reduce fitness (Vamosi and Lesack 2007) and body mass (Colegrave 1993; Vamosi 2005) at emergence. Indeed, within populations with high larval competition, females readily avoid laying additional eggs on seeds that have already been used (Fox and Messina 2018).

### 2.2 Thermal Evolution Lines and Assay Conditions

**FIGURE 1:**
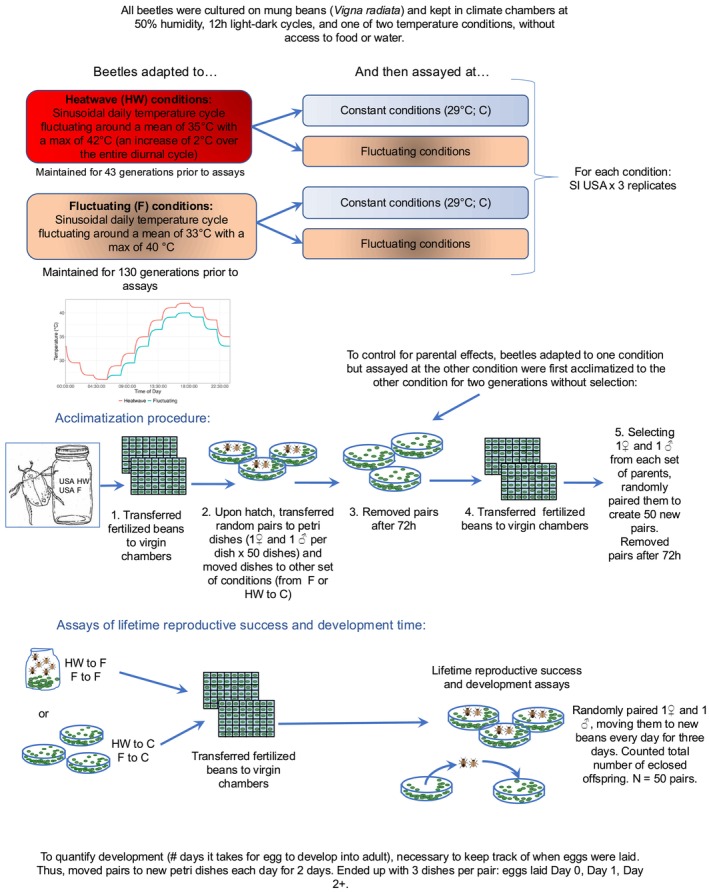
Experimental design for the thermal evolution lines, acclimatization procedure, and the reproductive success and development time assays. Figure by EB, CP, & EIC.

See Figure 1 for a detailed diagram. Before establishing treatment groups, we maintained a single ancestral population of SI USA beetles in two distinct Panasonic MLR‐351H (294 L) climate chambers. These chambers were maintained under identical humidity and light conditions but differed in their long‐term thermal regime. One population was maintained at a fluctuating thermal regime (hereafter, “Fluctuating”) for 130 generations (equivalent to evolving for ~8.1 years), whilst another was maintained for 43 generations (equivalent to evolving for ~2.7 years) at the same fluctuating thermal regime but experienced regular heatwaves (hereafter, “Heatwave”). The Heatwave population was derived from the Fluctuating population, which explains the different numbers of generations for each. For the “Fluctuating” thermal regime, the seed beetles were subjected to a daily temperature cycle consisting of 12 separate 2 h periods of constant temperature *T*_*i*_

$$T_{i}=T_{\text{mean}}+∆\text{Tsin}\left(\frac{i-12}{12}\pi \right),$$

where *T*_*mean*_ = 33°C, ∆ *T* = 7°C, and *i* = 0,1…. 11. This was a stepwise sinusoidal temperature cycle with *T*_max_ = 40°C and *T*_min_ = 26°C which mimics typical late spring condition in Southern India, where this population originated. For the “Heatwave” thermal regime, the beetles were exposed to an increase of 2°C over the entire diurnal cycle, reaching a *T*_max_ of 42°C, consistent with a heatwave in Southern India.

$${T^{h}}_{i}=T_{i}+2=T_{\text{mean}}+∆\text{Tsin}\left(\frac{i-12}{12}\pi \right)+2$$

Here, *T* ^h^_i_ is the temperature of the chamber during the heatwave regime at step *i*, over a period of 7 days starting at 6:00 am (the point of *T*_min_). To mimic the random character of the occurrences, the “Heatwave” regime was initiated on different days during the lifecycle of the beetles. Specifically, during each generation the “heatwave” regime was initiated a day earlier relative to the prior one. This was done so that over time, all developmental stages would experience the same number of heatwaves. In contrast, randomly applied heatwaves might have inadvertently impacted certain larval stages over others.

Prior to conducting experimental assays, we subdivided beetles from the two different thermal regimes into three replicates each (*n* = 6, 2 thermal regimes × 3 replicates. Note the unit of replication was *n* = 3 per treatment). For both thermal regimes, beetles from each of the three replicates were used to create a final six groups, which were either kept at their ancestral conditions or were acclimatized for two generations without selection to Constant conditions at 29°C (i.e., a common garden environment; Kawecki et al. 2012). In total this as done with a sample size of 50 beetle pairs each; *total n of treatments* = 12, 2 thermal regimes × 3 replicates × 2 environmental conditions. The assay conditions were as follows:

1. *Fluctuating–Constant*: Beetles that had evolved under Fluctuating conditions and then kept for two generations at Constant conditions of 29°C prior to assaying. This was done in order to control for any possible parental effects and to separate genetic adaptation from phenotypic plasticity and is a common practice in this species (Lind et al. 2014; Lymbery et al. 2020).
2. *Heatwave–Constant*: Beetles that had evolved under Heatwave conditions and then kept for two generations at Constant conditions of 29°C prior to assaying (see above).
3. *Heatwave–Fluctuating*: Beetles that had evolved under Heatwave conditions and remained in these conditions prior to assaying. Note that Fluctuating and Heatwave conditions are identical except when a periodic heatwave is happening. No heatwaves were applied during the two generations prior to or during the assay period, since the aim was to examine the fitness impacts of evolutionary changes that had occurred over many generations, not to measure the impact that a current heatwave might have at a particular developmental period (see below).
4. *Fluctuating–Fluctuating*: Beetles that had evolved under Fluctuating conditions and remained in these conditions prior to assaying.

For the Fluctuating‐Constant and the Heatwave‐Constant assays, the following steps were performed. First, we transferred beans containing fertilized eggs from jars into a 48‐well virgin chamber (aerated plastic culture plates with a separate well for each individual) with one bean allocated per well. The virgin chambers were then moved to the Constant climate chamber where they were kept at constant 29°C.

Once at least 50 males and 50 females from each virgin chamber had hatched, we randomly paired 1‐day‐old males and females and placed them in 60 mm petri dishes with 100 beans and returned these to the Constant chamber. *C. maculatus* females from this particular SI USA line lay a maximum of 100 eggs on beans in their lifetime (Berg and Maklakov 2012) and we ensured that each petri dish contained a sufficient number of beans to allow females to lay only one egg per bean, thereby minimizing larval competition. To further reduce larval competition, we removed adult beetle pairs from the petri dishes after 72 h (Berg and Maklakov 2012; Berg et al. 2019). Nineteen days later, before any offspring eclosed, we transferred up to 48 beans with eggs from each dish into separate, labeled 48‐well virgin chambers. One day following eclosion, we randomly selected and paired 50 females and 50 males (taking care not to pair full siblings) and placed them into 60‐mm petri dishes, each containing 80 beans. After 72 h, the adult males and females were removed from the petri dishes. We repeated this process for one additional generation: once again, before eclosion, beans containing fertilized eggs from each petri dish were transferred into separate 48‐well virgin chambers, one chamber per pair. We used the offspring that hatched out of these chambers for the development time (time from laying until hatching) and reproductive fitness assays (below).

For the Fluctuating‐Fluctuating and Heatwave‐Fluctuating treatments, we omitted the acclimatization steps, since the beetles were remaining at the same conditions in which they evolved and did not need to adjust to new conditions. As noted above, the Fluctuating and Heatwave regimes were identical except for the periodic blasts of elevated temperatures in the Heatwave treatment. To avoid any unintended effects of elevated heat exposure on larval development in the Heatwave beetles, we avoided applying heatwaves during the two generations prior to and during the assay period. To prepare for the assays, we directly transferred beans containing fertilized eggs into virgin chambers prior to hatching. For all assays, 1‐day‐old males and females were randomly selected.

### 2.3 Development Time and Reproductive Fitness Assays

Development time and fitness assays were conducted simultaneously. We paired 50 1‐day‐old male and female beetles and placed them into 60‐mm Petri dishes containing 70 beans. After 24 h, each pair was transferred together into a fresh 60‐mm petri dish containing 60 beans, while the initial dish was set aside in the same climate chamber. The following day, this procedure was repeated. The beetles were left in the third and last set of dishes, containing 50 beans, until their death. The number of beans in the dishes always exceeded the total number of eggs any female could lay in a single day at that age. The initial dishes for each pair were labeled as “Day 0,” the second dishes were labeled as “Day 1,” and the final set of dishes were labeled as “Day 2+.” 18 days after each dish was set up, before any new beetles could hatch, all beans with fertilized eggs were transferred into virgin chambers. These were then monitored daily to record the date of eclosion and the sex of all offspring. To calculate development time, we counted the number of days between the date that an egg was laid and the date the adult offspring eclosed. LRS was calculated as the total number of offspring that emerged from each pair, across all three reproductive days (and dishes; Days 0, 1, and 2+).

### 2.4 Statistical Analysis

All code was run using R v.4.4.2 (R Core Team 2021). Linear and generalized linear mixed were run initially in {glmmTMB} v.1.1.10 (Brooks et al. 2017; Magnusson et al. 2017) with residual diagnostics including detection of zero‐inflation and dispersion using {DHARMa} v.0.4.7 (Hartig 2017). Bias‐corrected estimated marginals means were then extracted from these models using {emmeans} v.1.10.7 (Lenth et al. 2019) corrected for by a multivariate *t* distribution (mvt). Data was imported, cleaned, and tidied using {readr} v.2.1.5, {tidyr} v.1.3.1, {dplyr} v1.1.4 (Wickham et al. 2019), {hablar} v.0.3.2 (Sjoberg 2020), {janitor} v.2.2.1 (Firke 2020), and {data.table} v.1.17.0 (Dowle et al. 2019). Data was then visualized using {ggplot2} v.3.5.1 (Wickham 2011). Heteroscedasticity was detected in the linear model for development time even after adding a predictor into the dispersion formula of the glmmTMB model so was instead run using {robustlmm} v.3.3‐1, which reduces the influence of outliers and fits models with robust estimation to reduce deviations from homoscedasticity (Koller 2016). All other packages used are found in the “renv” folder stored using the {renv} v.1.0.7 (Ushey 2023) package and contained within the session.info section of the code README within the repository.

For the model of lifetime reproductive success (or total reproduction across Days 0, 1, 2+) we added fixed categorical predictors of thermal regime (fluctuating and heatwave) in a two‐way interaction with assay environment (constant and fluctuating). A 12‐level random effect of population grouping (see above) was also added. The following model procedure was followed. Three models were initially fitted (a Poisson model, with and without an object‐level random effect, and a Conway‐Maxwell Poisson model) and tested for overdispersion and zero‐inflation. If significant zero‐inflation was still detected, we instead compared a zero‐inflated Poisson model (with and without an object‐level random effect) to a zero‐inflated Conway‐Maxwell Poisson model and compared these via Akaike Information Criterion (AIC; Akaike 1987). Lastly, if significant dispersion or heterogeneity of residuals was still detected after accounting for zero‐inflation, we performed model selection on parameters in the dispersion formula (including adding a 12‐level fixed factor of population group, as random effects are not allowed within the dispersion formula). Note, no model selection occurred on the main part of the model.

Development time was log‐transformed and ran in a Gaussian model with the same random (albeit with an additional random intercept of pair ID nested within the random factor of group to account for non‐independence) and fixed effects with interactions as above. Similar model selection took place on the dispersion formula of the model. However, even with this added dispersion parameter, significant heteroscedasticity was identified in residuals. This led to the use of a robust linear mixed effect model (see above). *p* values for the robust linear model were obtained using the {sjPlot} v. 2.8.17 package (Lüdecke 2013). The qualitative results did not vary between either methods.

## 3 Results

### 3.1 Lifetime Reproductive Success

**FIGURE 2:**
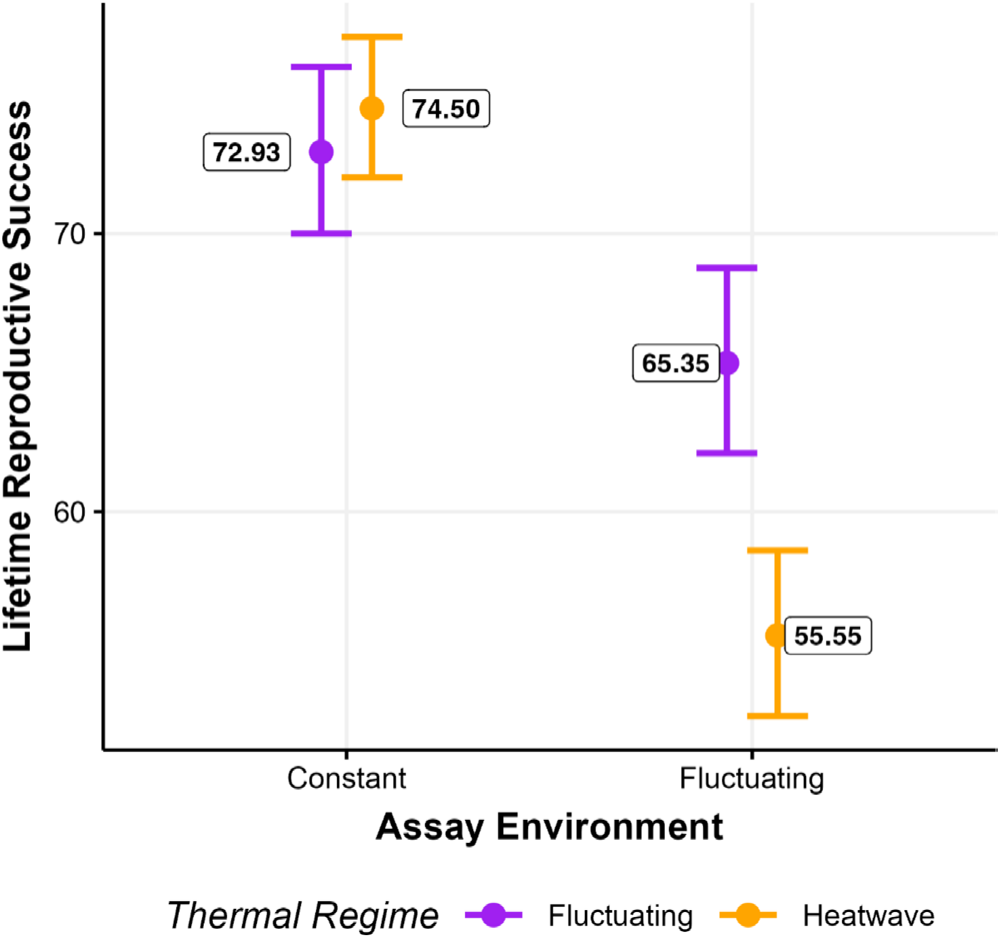
Lifetime reproduction success of individuals from heatwave (orange) and fluctuating (purple) thermal regimes assayed in constant (29°C) or fluctuating environments. Points with error bars represent mean values with accompanying 95% asymptotic confidence levels taken from the final model of lifetime reproductive success. Figure by EIC.

Lifetime reproductive success was significantly influenced by the interaction between thermal regime (Heatwave and Fluctuating) and assay environment (Constant and Fluctuating; Figure 2; *p* < 0.001; Tables S1 and S2). More specifically, when assayed under fluctuating conditions, individuals that evolved under heatwave conditions had reduced LRS compared to those that evolved under fluctuating conditions (*p* < 0.001; Table S3). This difference in LRS disappeared when assayed under constant conditions (*p* = 0.701; Figure 2: Table S3). For both thermal regimes, LRS under constant conditions was higher than fluctuating conditions (both *p* < 0.001; Figure 2; Table S3).

### 3.2 Development Time

**FIGURE 3:**
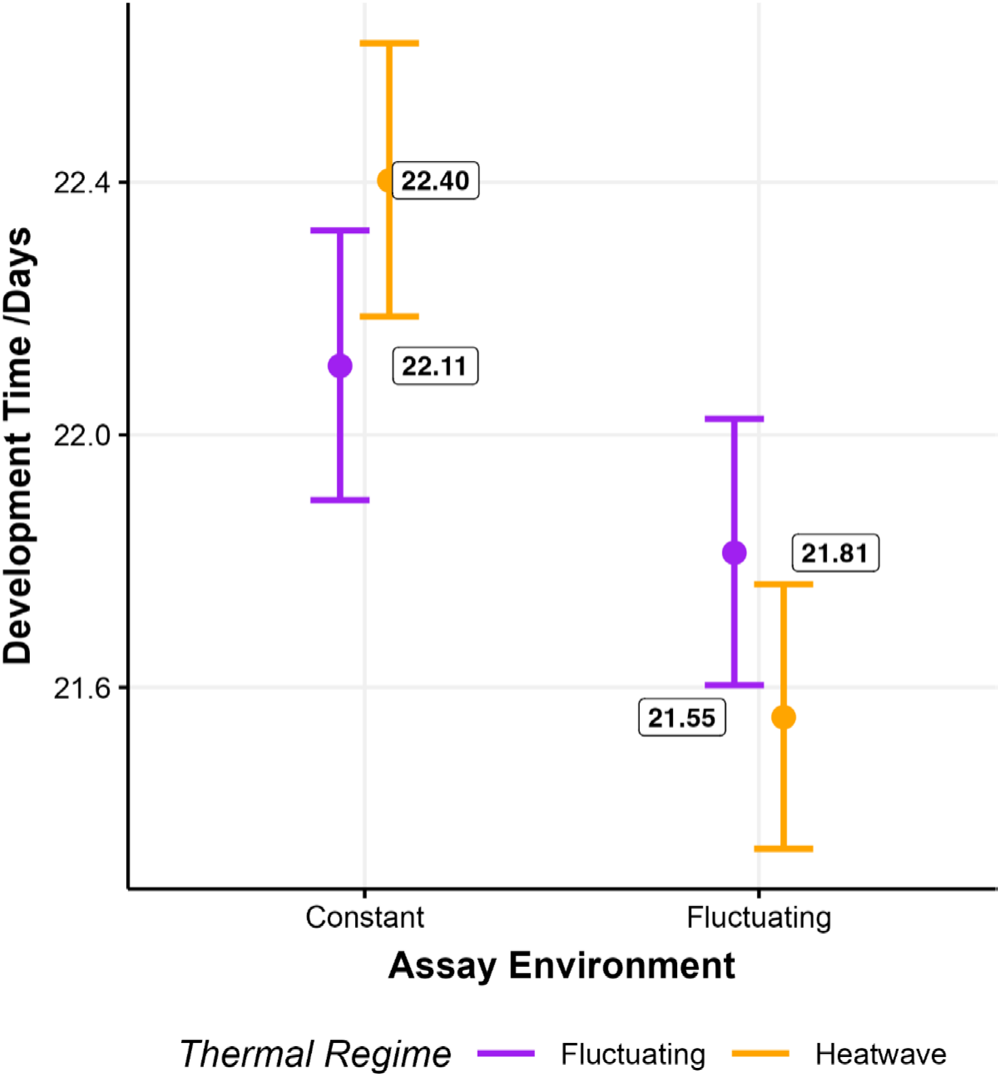
Development time (in days) of individuals from heatwave (orange) and fluctuating (purple) thermal regimes assayed in constant (29°C) or fluctuating environments. Points with error bars represent mean values with accompanying 95% asymptotic confidence levels taken from the final model of development time. Figure by EIC.

In a similar manner to LRS, the development time of individuals was influenced by the interaction between assay environment and thermal regime (*p* = 0.001; Figure 3; Table S4). However, estimated marginal means corrected for by a multivariate *t* distribution indicated no significant difference between Fluctuating and Heatwave regimes within the Constant or Fluctuating assay environments (*p* = 0.565 and 0.098, respectively; Figure 3; Table S5). However, there were differences between environments, with all individuals, regardless of thermal regime, taking longer to develop under constant conditions (*p =* 0.050 and < 0.001; Table S5), although this effect appeared larger for heatwave individuals (Figure 3).

## 4 Discussion

The aim of this study was to investigate the effects of long‐term heatwave exposure on seed beetles ( *C. maculatus* ) that had evolved under natural diel temperature fluctuations for multiple generations. Beetles adapted to fluctuating temperatures were exposed for 43 generations to periodic heatwaves and then assayed in either the ancestral fluctuating conditions or in a novel constant and benign environment. We had hypothesized that long‐term exposure to heatwaves would result in either (1) decreased fitness and impaired or delayed development relative to beetles that had not evolved with heatwaves (Sales et al. 2018, 2024), or conversely (2) improved resistance and tolerance to elevated temperatures (French et al. 2019; Ahrens et al. 2021; Xu et al. 2021). Additionally, we hypothesized that individuals from both the fluctuating and heatwave conditions would react in a similar manner when exposed to a novel constant and benign environment, similar to the previous experiment involving these experimental lines (Ivimey‐Cook et al. 2024).

Indeed, we found that individuals from populations exposed to regular heatwaves had significantly lower fitness (fewer eclosed offspring) than individuals from populations without this exposure. This negative effect of heatwave exposure aligns with previous research across a variety of species across both sexes (Sales et al. 2018; Martinet et al. 2021; Siegle et al. 2022; Ratz et al. 2024; Weaving et al. 2024). This decline is to be expected if there is negative effect of repeated and sustained elevation of heat shock proteins (Feder and Hofmann 1999; Sørensen et al. 2003; Siegle et al. 2022) and has been suggested to be closely linked to resource availability in the environment (Ketola et al. 2004). Among males, reproductive success might decline if heatwaves lead to reduced sperm count or motility or alter sperm morphology (Sales et al. 2021; Ratz et al. 2024). We may also expect this decrease if females suffered reproductive abnormalities, such as those found in the female tsetse fly after exposure to 30°C heat (Mellanby 1937; Weaving et al. 2024). Furthermore, given that every developmental stage experienced the same number of heatwaves over time, this difference in LRS could be a result of temporal misalignment between required phenotype and environment, or from the cost of evolving the necessary mechanisms to detect changes within the environment (such as heatwaves) and to produce appropriate phenotypes sufficiently rapidly (Burggren 2020; Hoffmann and Bridle 2021). To gain insight into the possible mechanisms underlying these differences in LRS, future studies should also include data on egg‐laying behaviors to determine whether females under heatwave conditions are laying fewer eggs, or whether more eggs are simply failing to develop.

However, it should be noted that the LRS between these two groups, although significant, is not markedly different (i.e., heatwave individuals exposed are still able to reproduce and are not functionally sterile), suggesting either some form of reproductive recovery or repair after prolonged heatwaves exposure (Ma et al. 2018; Sales et al. 2021), or the evolution of improved tolerance and resistance to elevated temperatures (French et al. 2019). Future work involving these long‐term evolution lines should therefore aim to compare the physiological differences, such as levels of expression of heat shock proteins or reproductive impairment, after one or multiple generations of heatwave exposure. Furthermore, to determine whether these recovery mechanisms occur within the lifetime of an individual or evolve across generations.

Although long‐term exposure to heatwaves appeared to have a negative impact on reproduction when the beetles were assayed at fluctuating conditions, we found that individuals from both fluctuating and heatwave conditions had increased reproductive fitness when assayed in a novel constant and benign 29°C environment. This is analogous to the previous study involving these experimental lines, Ivimey‐Cook et al. (2024), where two different strains of *C. maculatus* (SI USA and Leicester) that had evolved under diel fluctuations had higher LRS when assayed within a constant 29°C environment than when assayed under fluctuating conditions. This suggests that evolution in a fluctuating environment and under repeated heatwaves can select for individuals with a broadened thermal niche that are more robust after generations of mutation removal and selective mortality (Rankin and Sponaugle 2011; Ketola et al. 2013; Ivimey‐Cook et al. 2024). Also, if an individual has narrowed their thermal niche and postponed critical functions that only occur within a specific temperature window, as in the diel narrowing hypothesis (Kefford et al. 2022), then reproductive performance should increase when individuals are placed within a constant and benign environment (Gilchrist 1995; Kefford et al. 2022). This is an important result, which further highlights how evolution under fluctuating environments can lead to an improved response to short‐term changes in environment, regardless of heatwave exposure.

Lastly, we found no negative effect of heatwave exposure on individual development time. Beetles from both thermal backgrounds developed faster in fluctuating environments, where the average mean temperature was 4°C hotter than constant conditions. This is similar to the result found in the previous experiment involving the same thermal conditions (Ivimey‐Cook et al. 2024), with the fastest development occurring within a fluctuating environment. The lack of a detrimental effect of long‐term heatwave exposure on development time is counter to what has been found previously. Some studies have shown a positive effect of elevated temperature exposure, such as in the parasitoid *Cotesia glomerata* , which developed twice as quickly in higher temperatures (Chen et al. 2019). Other studies have shown negative effects, for instance in the solitary bee *Osmia lignaria*, which developed more slowly under 37°C heatwave conditions than the no‐heatwave control (Melone et al. 2024). However, in both of these cases, individuals were exposed to just one generation of heatwave rather than testing them after multiple (in our case 43) generations. Therefore, we may see a reduction in effect over time when individuals are exposed to more repeated heatwaves and become more resilient to environmental change (Ahrens et al. 2021; Xu et al. 2021).

In conclusion, we found that populations evolving under repeated, long‐term heatwave exposure within naturally fluctuating environments experience decreased, although not markedly reduced, reproductive success indicative of a minor cost to adaptation. However, they are also seemingly able to cope with rapid environmental change, reaching maximal performance when conditions suddenly become more benign. Overall, this study highlights the importance of considering long‐term diel fluctuations together with multi‐generational exposure to heatwaves in order to better understand natural population responses to climatic warming.

## Author Contributions

**Edward R. Ivimey‐Cook:** conceptualization (equal), formal analysis (lead), visualization (lead), writing – original draft (lead), writing – review and editing (equal). **Sarah Glavan:** investigation (supporting). **Sophie Bricout:** investigation (equal), data curation (lead), methodology (supporting). **Claudio Piani:** investigation (supporting), methodology (supporting), writing – review and editing (supporting). **Elena C. Berg:** conceptualization (equal), funding acquisition (lead), methodology (lead), project administration (lead), investigation (equal), supervision (lead), writing – original draft (supporting), writing – review and editing (equal).

## Funding

This work was supported by The American University of Paris.

## Conflicts of Interest

The authors declare no conflicts of interest.

## Supporting information


**Table S1:** Model selection for the best fitting model for lifetime reproductive success (LRS). Showing the top five best fitting models ranked by AIC. C‐M‐P stands for Conway‐Maxwell Poisson. The best model is denoted in bold.
**Table S2:** Output of the final model for LRS. Significant predictors are bolded.
**Table S3:** Pairwise interactions of estimated marginal means from the final model of LRS. Significant comparisons are bolded. Columns denoted are the environment, the thermal regime, the contrast being compared, the ratio (which denotes how different the two contrasts are from each other, with a value of one meaning now difference), the lower confidence limits, the upper confidence limits, the *z* value test statistics, and lastly, the *p* value.
**Table S4:** Output of the final model for development time (robust linear mixed model). Significant predictors are bolded.
**Table S5:** Pairwise interactions of estimated marginal means from the final model of development time. Significant comparisons are bolded. Columns denoted are the environment, the thermal regime, the contrast being compared, the ratio (which denotes how different the two contrasts are from each other, with a value of one meaning now difference), the lower confidence limits, the upper confidence limits, the *z* value test statistics, and lastly, the *p* value.

## Data Availability

Data and code are available on Zenodo at https://doi.org/10.5281/zenodo.19680817.
