# Supplementary material for: Long‐Term Evolution Under Heatwave Conditions in the Seed Beetle, Callosobruchus maculatus
Source: Ecol Evol. 2026 Apr 23;16(4):e73562. doi: 10.1002/ece3.73562 (PMC13105836; doi:10.1002/ece3.73562)
Supplement: Supplementary file 1 — Table S1: Model selection for the best fitting model for lifetime reproductive success (LRS). Showing the top five best fitting models ranked by AIC. C‐M‐P stands for Conway‐Maxwell Poisson. The best model is denoted in bold. Table S2: Output of the final model for LRS. Significant predictors are bolded. Table S3: Pairwise interactions of estimated marginal means from the final model of LRS. Significant comparisons are bolded. Columns denoted are the environment, the thermal regime, the contrast being compared, the ratio (which denotes how different the two contrasts are from each other, with a value of one meaning now difference), the lower confidence limits, the upper confidence limits, the z value test statistics, and lastly, the p value. Table S4: Output of the final model for development time (robust linear mixed model). Significant predictors are bolded. Table S5: Pairwise interactions of estimated marginal means from the final model of development time. Significant comparisons are bolded. Columns denoted are the environment, the thermal regime, the contrast being compared, the ratio (which denotes how different the two contrasts are from each other, with a value of one meaning now difference), the lower confidence limits, the upper confidence limits, the z value test statistics, and lastly, the p value. [file ECE3-16-e73562-s001.docx]

**Supplementary material: Long-term evolution under heatwave conditions in the seed beetle, *Callosobruchus maculatus***

**Table S1.** Model selection for the best fitting model for lifetime reproductive success (LRS). Showing the top 5 best fitting models ranked by AIC. C-M-P stands for Conway-Maxwell Poisson. The best model is denoted in bold.

| Family | Formula | zi | dispersion | AIC | *df* |
| --- | --- | --- | --- | --- | --- |
| **C-M-P** | **regime + env + regime:env + (1 \| group)** | **~1** | **~group** | **4965.18** | **18** |
| C-M-P | regime + env + regime:env + (1 \| group) | ~1 | ~env | 4987.24 | 8 |
| C-M-P | regime + env + regime:env + (1 \| group) | ~1 | ~env + regime | 4988.94 | 9 |
| C-M-P | regime + env + regime:env + (1 \| group) | ~1 | ~env * regime | 4989.70 | 10 |
| C-M-P | regime + env + regime:env + (1 \| group) | ~1 | ~1 | 5002.79 | 7 |

**Table S2.** Output of the final model for LRS. Significant predictors are bolded.

| ***Predictors*** | ***Estimates*** | ***std. Error*** | ***Statistic*** | ***p*** |
| --- | --- | --- | --- | --- |
| (Intercept) | 4.29 | 0.02 | 260.28 | **<0.001** |
| regime [Heatwave] | 0.02 | 0.02 | 1.00 | 0.319 |
| env [Fluctuating] | -0.11 | 0.03 | -4.18 | **<0.001** |
| regime [Heatwave] × env [Fluctuating] | -0.18 | 0.04 | -5.02 | **<0.001** |
| ***Dispersion Component*** | | | | |
| (Intercept) | 0.84 | 0.21 | 4.09 | **<0.001** |
| groupF-C-2 | 0.01 | 0.29 | 0.03 | 0.978 |
| groupF-C-3 | 0.67 | 0.30 | 2.25 | **0.024** |
| groupF-F-1 | 0.73 | 0.29 | 2.50 | **0.013** |
| groupF-F-2 | 0.21 | 0.29 | 0.74 | 0.462 |
| groupF-F-3 | 0.84 | 0.29 | 2.88 | **0.004** |
| groupH-C-1 | -0.01 | 0.29 | -0.03 | 0.973 |
| groupH-C-2 | 0.52 | 0.29 | 1.77 | 0.076 |
| groupH-C-3 | -0.61 | 0.29 | -2.10 | **0.036** |
| groupH-H-1 | 0.30 | 0.29 | 1.03 | 0.304 |
| groupH-H-2 | 0.25 | 0.29 | 0.87 | 0.384 |
| groupH-H-3 | 1.24 | 0.30 | 4.10 | **<0.001** |
| ***Zero-Inflated Component*** | | | | |
| (Intercept) | -5.01 | 0.50 | -9.95 | **<0.001** |
| ***Random Effects*** | | | | |
| σ^2^ | NA | | | |
| τ_00_ _group_ | 0.00 | | | |
| N _group_ | 12 | | | |
| Observations | 600 | | | |

**Table S3.** Pairwise interactions of estimated marginal means from the final model of LRS. Significant comparisons are bolded. Columns denoted are the environment, the thermal regime, the contrast being compared, the ratio (which denotes how different the two contrasts are from each other, with a value of 1 meaning now difference), the lower confidence limits, the upper confidence limits, the z value test statistics, and lastly, the *p* value.

| **Env** | **Regime** | **Contrast** | **Ratio** | ***std. Error*** | **LCL** | **UCL** | **z** | **p** |
| --- | --- | --- | --- | --- | --- | --- | --- | --- |
| Constant | . | Fluctuating / Heatwave | 0.979 | 0.021 | 0.929 | 1.03 | -0.996 | 0.701 |
| **Fluctuating** | **.** | **Fluctuating / Heatwave** | **1.176** | **0.035** | **1.094** | **1.265** | **5.475** | **<0.001** |
| **.** | **Fluctuating** | **Constant / Fluctuating** | **1.116** | **0.029** | **1.047** | **1.190** | **4.182** | **0.0001** |
| **.** | **Heatwave** | **Constant / Fluctuating** | **1.341** | **0.034** | **1.260** | **1.427** | **11.54** | **<0.001** |

**Table S4.** Output of the final model for development time (robust linear mixed model). Significant predictors are bolded.

| ***Predictors*** | ***Estimates*** | ***std. Error*** | ***Statistic*** | ***p*** |
| --- | --- | --- | --- | --- |
| (Intercept) | 3.10 | 0.00 | 797.81 | **<0.001** |
| regime [Heatwave] | 0.01 | 0.01 | 2.41 | **0.016** |
| env [Fluctuating] | -0.01 | 0.01 | -2.45 | **0.014** |
| regime [Heatwave] × env [Fluctuating] | -0.03 | 0.01 | -3.24 | **0.001** |
| **Random Effects** | | | | |
| σ^2^ | 0.00 | | | |
| τ_00_ _id:group_ | 0.00 | | | |
| τ_00_ _group_ | 0.00 | | | |
| N _id_ | 596 | | | |
| N _group_ | 12 | | | |
| Observations | 39876 | | | |

**Table S5.** Pairwise interactions of estimated marginal means from the final model of development time. Significant comparisons are bolded. Columns denoted are the environment, the thermal regime, the contrast being compared, the ratio (which denotes how different the two contrasts are from each other, with a value of 1 meaning now difference), the lower confidence limits, the upper confidence limits, the z value test statistics, and lastly, the *p* value.

| ***Env*** | ***Regime*** | ***Contrast*** | ***Ratio*** | ***std. Error*** | ***LCL*** | ***UCL*** | ***z*** | ***p*** |
| --- | --- | --- | --- | --- | --- | --- | --- | --- |
| Constant | . | Fluctuating / Heatwave | 0.987 | 0.005 | 0.974 | 1.000 | -2.405 | 0.057 |
| Fluctuating | . | Fluctuating / Heatwave | 1.012 | 0.006 | 0.999 | 1.026 | 2.183 | 0.098 |
| **.** | **Fluctuating** | **Constant / Fluctuating** | **1.014** | **0.006** | **1.000** | **1.027** | **2.454** | **0.050** |
| **.** | **Heatwave** | **Constant / Fluctuating** | **1.039** | **0.006** | **1.026** | **1.054** | **7.035** | **0.000** |
